# Molecular phylogeny confirms the subspecies delineation of the Malayan Siamang (*Symphalangussyndactyluscontinentis*) and the Sumatran Siamang (*Symphalangussyndactylussyndactylus*) based on the hypervariable region of mitochondrial DNA

**DOI:** 10.3897/BDJ.12.e120314

**Published:** 2024-04-25

**Authors:** Nur Hartini Sariyati, Muhammad Abu Bakar Abdul-Latiff, Nor Rahman Aifat, Abd Rahman Mohd-Ridwan, Nur Azimah Osman, Kayal Vizi Karuppannan, Eddie Chan, Badrul Munir Md-Zain

**Affiliations:** 1 Faculty of Applied Sciences and Technology, Universiti Tun Hussein Onn Malaysia (Pagoh Campus), 84600, Muar, Johor, Malaysia Faculty of Applied Sciences and Technology, Universiti Tun Hussein Onn Malaysia (Pagoh Campus) 84600, Muar, Johor Malaysia; 2 Department of Biological Sciences and Biotechnology, Faculty of Science and Technology, Universiti Kebangsaan Malaysia, 43600, Bangi, Selangor, Malaysia Department of Biological Sciences and Biotechnology, Faculty of Science and Technology, Universiti Kebangsaan Malaysia, 43600 Bangi, Selangor Malaysia; 3 Faculty of Tropical Forestry, Universiti Malaysia Sabah, 88400, Kota Kinabalu, Sabah, Malaysia Faculty of Tropical Forestry, Universiti Malaysia Sabah, 88400 Kota Kinabalu, Sabah Malaysia; 4 Centre for Pre-University Studies, Universiti Malaysia Sarawak, 94300, Kota Samarahan, Sarawak, Malaysia Centre for Pre-University Studies, Universiti Malaysia Sarawak, 94300 Kota Samarahan, Sarawak Malaysia; 5 Faculty of Applied Sciences, Universiti Teknologi Mara Negeri Sembilan, 72000, Kuala Pilah, Negeri Sembilan, Malaysia Faculty of Applied Sciences, Universiti Teknologi Mara Negeri Sembilan, 72000 Kuala Pilah, Negeri Sembilan Malaysia; 6 National Wildlife Forensic Laboratory (NWFL), Department of Wildlife and National Parks (PERHILITAN), 56100, Kuala Lumpur, Malaysia National Wildlife Forensic Laboratory (NWFL), Department of Wildlife and National Parks (PERHILITAN), 56100 Kuala Lumpur Malaysia; 7 Genting Nature Adventure, Resorts World Awana Hotel, 69000, Genting Highlands, Pahang, Malaysia Genting Nature Adventure, Resorts World Awana Hotel 69000, Genting Highlands, Pahang Malaysia

**Keywords:** *Symphalangussyndactylus*, siamang, small ape, Hylobatidae, phylogenetic

## Abstract

Siamangs (*Symphalangussyndactylus*) are native to Peninsular Malaysia, Sumatra and southern Thailand and their taxonomical classification at subspecies level remains unclear. Morphologically, two subspecies were proposed as early as 1908 by Thomas namely *Symphalangus* *s.syndactylus* and *Symphalanguss.continentis.* Thus, this study aims to clarify the Siamang subspecies status, based on mtDNA *D*-loop sequences. Faecal samples were collected from wild Siamang populations at different localities in Peninsular Malaysia. A 600-bp sequence of the mitochondrial *D*-loop region was amplified from faecal DNA extracts and analysed along with GenBank sequences representing *Symphalangus* sp., *Nomascus* sp., *Hylobates* sp., *Hoolock* sp. and outgroups (*Pongopygmaeus*, *Macacafascicularis* and *Papiopapio*). The molecular phylogenetic analysis in this study revealed two distinct clades formed by *S.s.syndactylus* and *S.s.continentis* which supports the previous morphological delineation of the existence of two subspecies. Biogeographical analysis indicated that the Sumatran population lineage was split from the Peninsular Malaysian population lineage and a diversification occurrred in the Pliocene era (~ 3.12 MYA) through southward expansion. This postulation was supported by the molecular clock, which illustrated that the Peninsular Malaysian population (~ 1.92 MYA) diverged earlier than the Sumatran population (~ 1.85 MYA). This is the first study to use a molecular approach to validate the subspecies statuses of *S.s.syndactylus* and *S.s.continentis*. This finding will be useful for conservation management, for example, during Siamang translocation and investigations into illegal pet trade and forensics involving Malayan and Sumatran Siamangs.

## Introduction

**Figure 1.**
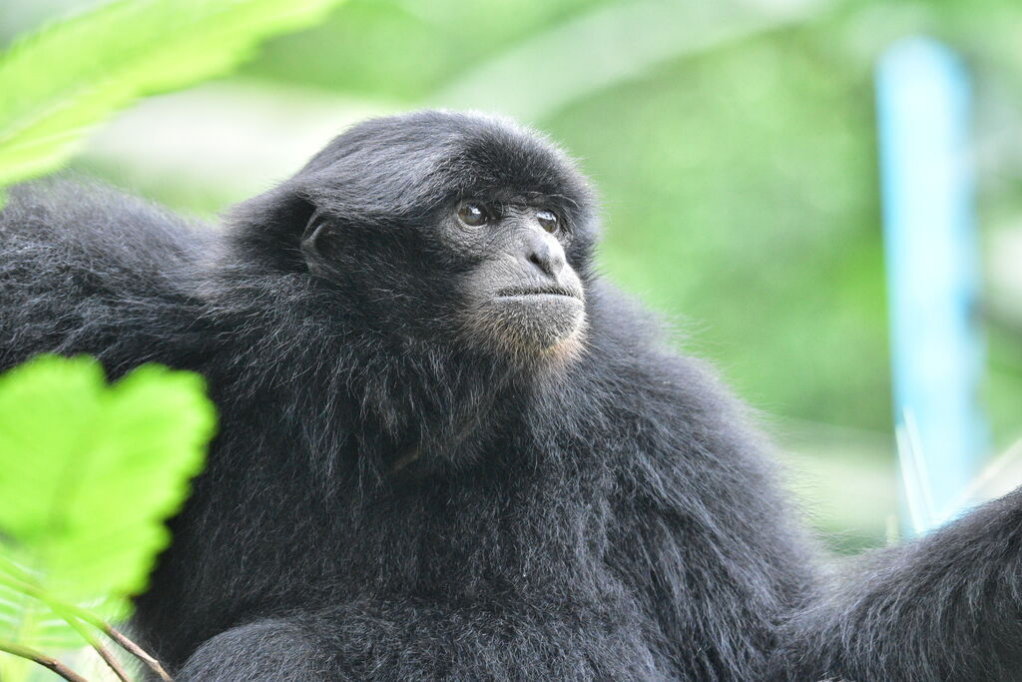
Siamang of Peninsular Malaysia.

The Siamang (*Symphalangussyndactylus*) is a small ape species with declining numbers and, thus, it has been classified as an endangered species and is included in the Red List of the International Union for Conservation of Nature (Nijman et al. 2020). The Siamang (Fig. 1) is distributed in Peninsular Malaysia and Sumatra, Indonesia (Brandon-Jones et al. 2004, Roos et al. 2014, Md-Zain et al. 2021,

Md-Zain et al. 2022), with a few populations living in the central and western parts of the Bala Forest of southern Thailand (Thong-aree 2000). It was once classified as a subgenus of *Hylobates* (Kloss 1929) until it was elevated to the full genus level under the name *Symphalangus*. Later, gibbons comprise two genera, namely, *Symphalangus* and *Hylobates* (Napier and Napier 1967). Primatologists recognise the following four genera of Hylobatidae using morphological (Groves 2001), behavioural (Geissmann 2002) and molecular criteria (Chivers 2013, Roos 2016): *Hoolock, Symphalangus, Hylobates* and *Nomascus*. *Symphalangussyndactylus*, first described in 1821, is the sole representative of the genus *Symphalangus* (Raffles 1821). Subsequently, two subspecies of Siamangs, namely, the Sumatran Siamang and Peninsular Malaysian Siamang, were described by Thomas (1908) on the basis of their skull appearance. The classification gained support from other researchers (Hooijer 1960, Frisch 1967), who provided more detailed information on the morphological traits of *S.s.syndactylus* and *S.s.continentis*. Mootnick (2006) observed Siamangs in a rescue centre and, similar to previous studies, he detected some morphological differences between *S.s.syndactylus* and *S.s.continentis*, particularly on the nose and toe structures.

The sequence of the mitochondrial DNA (mtDNA) *D*-loop region has been widely used to examine the origin and genetic relationships of various mammals (Kamaluddin et al. 2018, Abdul-Latiff et al. 2019, Karuppannan et al. 2019, Abdul-Latiff and Md-Zain 2021). Whittaker et al. (2007) were the first to demonstrate the effectiveness of the *D*-loop region in reconstructing the phylogeny of *Hylobates*. Furthermore, the availability of complete mitochondrial genomes has resolved the branches amongst the members of Hylobatidae (Matsudaira and Ishida 2010). For instance, the separation of *Nomascus* as a sister clade of *Hylobates* and *Symphalangus* (excluding *Hoolock*) has been strongly supported by mtDNA studies (Chan et al. 2010, Matsudaira and Ishida 2010). Fan et al. (2017) proved that the combined use of genomic and morphological analyses is effective in resolving taxonomic issues up to the species level. In general, mitochondrial gene sequences have been useful in understanding the genetic relationships amongst isolated primate populations. For example, Thinh et al. (2010) used the mitochondrial cytochrome *b* gene sequence to clarify that *H.albibarbis* is more closely related to *H.agilis* in Sumatra than to other geographically closer populations (*H.muelleri, H. funereus* and *H.abbotti*) in Borneo. In addition, the successful use of genetic markers resolved the taxonomical issue up to the subspecies level as proven by Roos et al. (2008). New silvered langur subspecies (*Trachypithecuscristatusselangorensis*) was successfully identified by cytochrome b sequencing. Thus, choosing mtDNA as a genetic marker in this study probably yielded a successful result in distinguishing the two populations of Siamang.

Previous research efforts on the Malayan Siamang have concentrated mostly on behavioural enticement and ecology (Chivers et al. 1975, Gittins and Raemaekers 1980). Thus far, no research has concentrated on molecular studies. Taxonomically, Roos (2016) has stated that Siamangs can be reliably classified up to the species level, but subspecies classification lacks strong support. In terms of the two subspecies in Peninsular Malaysia and Sumatra, Indonesia, molecular data for the Malaysian subspecies are lacking. Molecular evidence is needed to confirm the subspecies classification first proposed by Thomas (1908) using morphological data. Molecular data are important to verify the morphologically based taxonomic classification of Siamangs at the subspecies level (Abdul-Latiff et al. 2014, Mohd-Yusof et al. 2020). Subspecies information is important for conservation efforts to avoid primate introgression in captivity. For example, during translocation, the correct Siamang subspecies must be matched to their proper habitat to ensure their survival. In addition, clarifying the systematics of Siamangs provides important phylogenetic information needed to understand the evolutionary history of these species. Thus, the aim of this research is to analyse the genetic relationships amongst subspecies of Siamangs from populations in Peninsular Malaysia and Sumatra, along with other members of Hylobatidae.

## Material and methods

### Sample collection, DNA extraction and polymerase chain reaction (PCR)

Faecal samples (Table 1) were kept in absolute ethanol (99%) and stored at −20°C to preserve the DNA in the faces (Aifat et al. 2021). DNA was extracted from 0.5–1.0 g of faeces using the InnuPREP Stool DNA Kit (Analytik Jena, Germany) according to manufacturer’s protocol (Md-Zain et al. 2019). An approximately 600-bp region of the *D*-loop gene was amplified by PCR using a species-specific primer pair (GIBDLF3 5′-CTTCACCCTCAGCACCCA AAGC-3′ and GIBDLR4 5’-GGGTGATAGGCCTGTGATC-3’), as described by Whittaker et al. (2007). Amplifications were carried out using MyTaq ™ Red Mix from Bioline in a total reaction volume of 25 µl consisting of 12.5 µl of MyTaq™ Red Mix, 3.0 µl of DNA template, 1.0 µl of each 20 µM primer and 7.5 µl ddH_2_O. The thermal cycling programme consisted of a predenaturation at 95°C for 3 min, followed by 30 cycles of denaturation at 95°C for 15 s, annealing at 50°C for 30 s and elongation at 72°C for 10 s and final elongation at 72°C for 10 min. The PCR products were electrophoresed using 1.5% agarose gel in 1× TAE buffer.

Amplified DNA was purified using the InnuPREP Double Pure Kit (Analytik Jena). The purified PCR product was sequenced by Apical Scientific Sdn. Bhd., Malaysia and the resulting sequences were edited using BioEdit software v.7.2.6.1 (Alzohairy 2011). The sequence chromatogram was visually proofread,and, then, the validity of the sequence was checked against other GenBank sequences using BLAST to evaluate quality and degree of match of our DNA sequences to the expected species and gene locus.

### Phylogenetic and haplotype analysis

The phylogenetic trees were constructed using a number of methods for a robust comparison: distance-based method (Neighbour-joining), character-based method (Maximum Parsimony), likelihood (Maximum Likelihood), Bayesian Inference and molecular clock estimation. Approximately 348 bp was used for phylogenetic and haplotype analyses. Of approximately 255 variable sites from sequence analyses, 217 were parsimony informative. The Neighbour-joining (NJ) and Maximum Parsimony (MP) trees were constructed using Mega X software (Kumar et al. 2018) and Maximum Likelihood (ML) was constructed using PAUP 4.0b10 (Kimura 1980). The tree was constructed with 1000 bootstrap replications to obtain the bootstrap confidence level, which estimates the robustness of the tree topologies. The Bayesian tree was constructed using an appropriate substitution model for *D*-loop sequences, which was selected by hLRT in ModelTest 3.7. The best model was HKY + G, with a gamma shape parameter of 0.7293 and base frequencies of 0.3495 for adenosine, 0.3136 for cytosine, 0.1103 for guanine and 0.2346 for thymine. A metropolis-coupled Markov Chain Monte Carlo model was run with 10 million generations and the tree was sampled every 100 generations.

The molecular clock was constructed using BEAUti 1.8.4 and BEAST 1.8.4 (Drummond et al. 2012). We defined Siamang datasets in BEAST 1.8.4, an outgroup dataset (*Papiopapio*, *Macacafascicularis* and *Pongopygmaeus*) was used as a calibration point and the other dataset included members of the family Hylobatidae (*Nomascus, Hoolock, Symphalangus* and *Hylobates*). The molecular divergence phylogenetic tree was constructed using the uncorrelated log-normal relaxed clock model (Drummond et al. 2006) and a Yule model before estimating the substitution rate for all nodes in the tree with uniform priors on the mean (0, 100) and standard deviation (0, 10). Published dates with a mean of 33.65 Ma and standard deviation of 3.45 Ma ([CI]: 30–37.1 Ma) were utilised for *M. fascicularis and P. papio* (Israfil et al. 2011). The tree was run for 10 million generations and sampled every 100 generations, with 10% of the initial trees discarded as burn-in. Tracer version 1.5 was used to assess the estimated sample size (ESS) from the log files produced by BEAST and the ESS of all parameters exceeded 200. The maximum-clade-credibility tree topologies were calculated from the posterior distribution and TreeAnnotator version 1.8.4 was employed to summarise the trees and view them in FigTree v.1.4.3. Finally, DnaSP v.4.0 was used to determine the number of haplotypes, haplotype diversity and segregation sites in the population (Rozas et al. 2003) and the relationships between haplotypes were illustrated in the form of minimum-spanning network (MSN) through Network 4.6.1.2.

## Data resources

Nine faecal samples were used in this study. These samples were collected from Peninsular Malaysia and included six samples of *S.syndactylus* (collected from Fraser’s Hill and Genting Highlands, Pahang) and three samples of *H.lar* (obtained from Melaka and Johor) as in the Table 1. The DNA sequences were deposited in GenBank of NCBI (MW117116-MW117124). Thirty-five *D*-loop region sequences representing Sumatran Siamangs, other Hylobatidae species (*Hoolock, Nomascus* and *Hylobates*) from various localities and three outgroup sequences including *Papiopapio*, *Macacafascicularis* and *Pongopygmaeus* (Table 1), were retrieved from GenBank.

## Results

### Neighbour-joining tree

**Figure 2.**
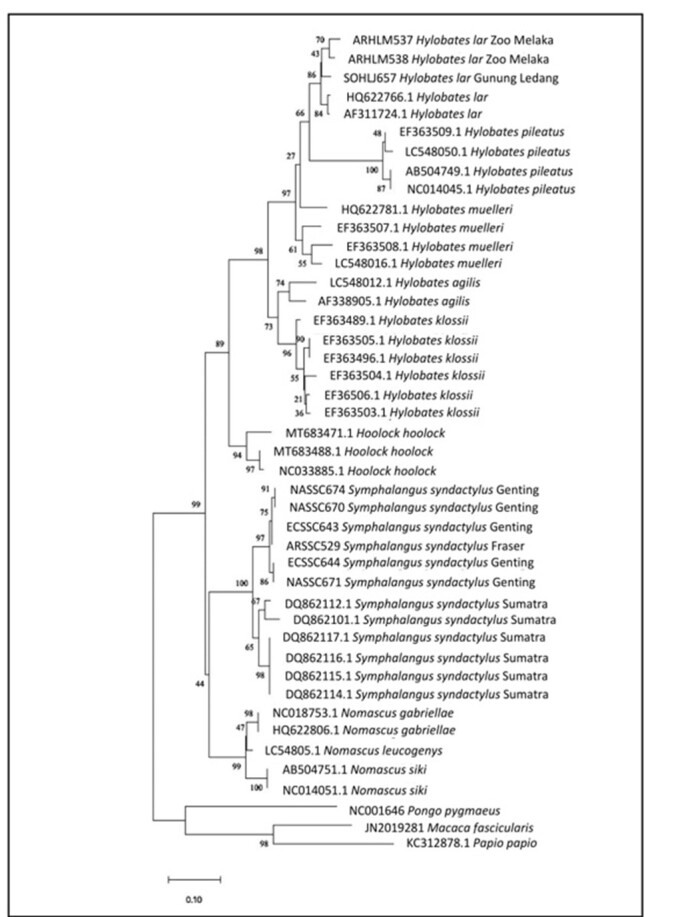
Neighbour-joining (NJ) phylogeny tree. Bootstrap values are indicated above the branch.

The NJ phylogeny tree (Fig. 2) was generated using the Kimura-2 parameter with 1000 bootstrap replications. All Siamang samples remained in the monophyletic clade with a 100% bootstrap value. Siamangs formed two clades. The first clade represented the monophyletic group of Siamangs from Peninsular Malaysia supported by a 97% bootstrap value. Amongst this clade, ECSS644 and NASSC671 formed a monophyletic subclade with 86% bootstrap support that branched out from other Peninsular Siamangs. The remaining Peninsular Siamangs formed another monophyletic subclade supported by a high bootstrap value (97%). Siamangs from Fraser’s Hill (ARSSC529) had the closest relationship with Siamangs from Genting Highlands (ECSSC643), although other individuals from Genting Highlands (ECSSC643, ECSSC644, ECSSC674, NASSC670 and NASSC671) were geographically sympatric.

The second clade depicted the separation of the Siamang monophyletic subclade formed by DQ862110 and DQ862112.1 from other Sumatran Siamangs distinguished by a 67% bootstrap value. The remaining individuals (DQ862114.1, DQ862115.1, DQ862116.1 and DQ862117.1) formed a monophyletic subclade supported by a high bootstrap value of 98%. The tree topology also depicted two major clades: *Hylobates* + *Hoolock* (supported with an 89% bootstrap value) and *Symphalangus* + *Nomascus* (supported by a 44% bootstrap value). The *Nomascus* clade was formed by *N.siki*, *N.leucogenys* and *N.gabriellae* with a 99% bootstrap value. The *Hoolock* clade supported by a 94% bootstrap value was represented by *H.hoolock*. Within clade *Hylobates*, further subclades were defined. The first subclade characterised the *klossii*–*agilis* relationships supported by a 73% bootstrap value. The second subclade represented three *Hylobates* species, which were *H.muelleri*, *H.pileatus* and *H.lar*, supported by a high bootstrap value of 97%. *H.muelleri* was the first to diverge from two other species, followed by splitting of *H.pileatus* from *H.lar* population.

### Maximum parsimony tree

**Figure 3.**
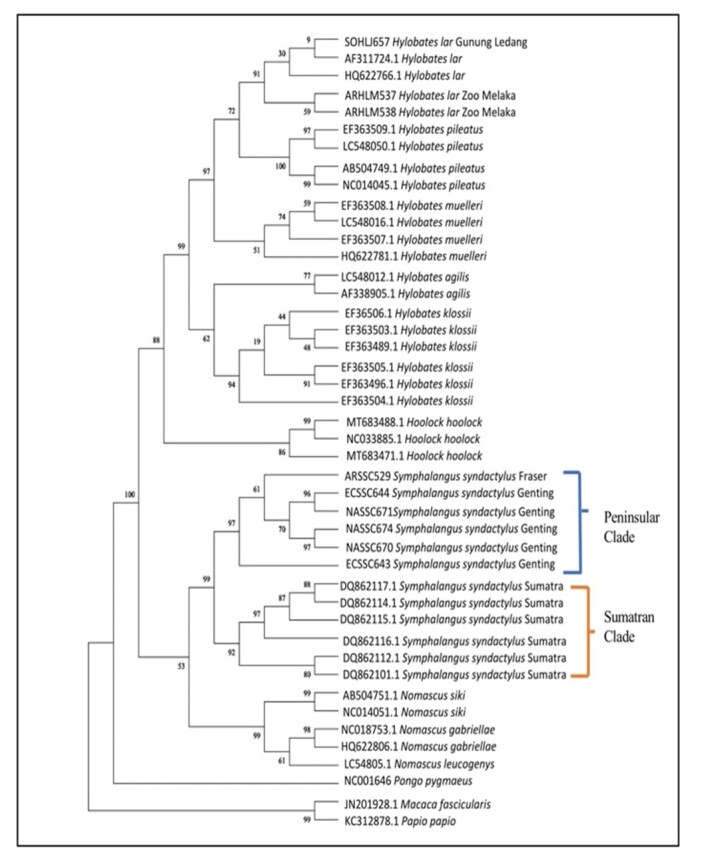
Maximum parsimony (MP) phylogeny tree. Bootstrap values are indicated above the branch.

The MP tree (Fig. 3) agreed with all Siamang groups in the same clade supported by a 99% bootstrap value. The differentiation between Peninsular and Sumatran Siamangs shown by MP analysis was parallel to the NJ tree, except for the subclade formed by Siamang members. The two clades structured within *Symphalangus* represented the Peninsular and Sumatran clades, which were supported by 97% and 92% bootstrap values, respectively. In the Peninsular Siamang clade, ECSSC643 was the earliest to branch out from the clade comprising ECSS644, ARSSC529, NASSC670, NASSC671 and NASSC674 (supported by a 61% bootstrap value). In the Sumatran clade, the branch topology within the members was equal to that of the NJ tree, but was supported with a higher bootstrap value. An 80% bootstrap value had grouped DQ862112.1 and DQ862101.1 together and formed a sister subclade to the remaining Sumatran Siamangs that the subclade supported by 97%. The topology pattern of hylobatids positively reflected the NJ tree where *Nomascus* formed a monophyletic clade with *Symphalangus* (53% bootstrap value) and the *Hoolock* clade together with *Hylobates* (88% bootstrap value). Each genus further branched into its own monophyletic clade: *Nomascus*, *Symphalangus*, *Hoolock* and *Hylobates* supported by 99%, 99%, 86% and 99% bootstrap values, respectively. For the *Hylobates* clade, *H.klossii* and *H.agilis* remained as the basal species.

### Maximum Likelihood tree

**Figure 4.**
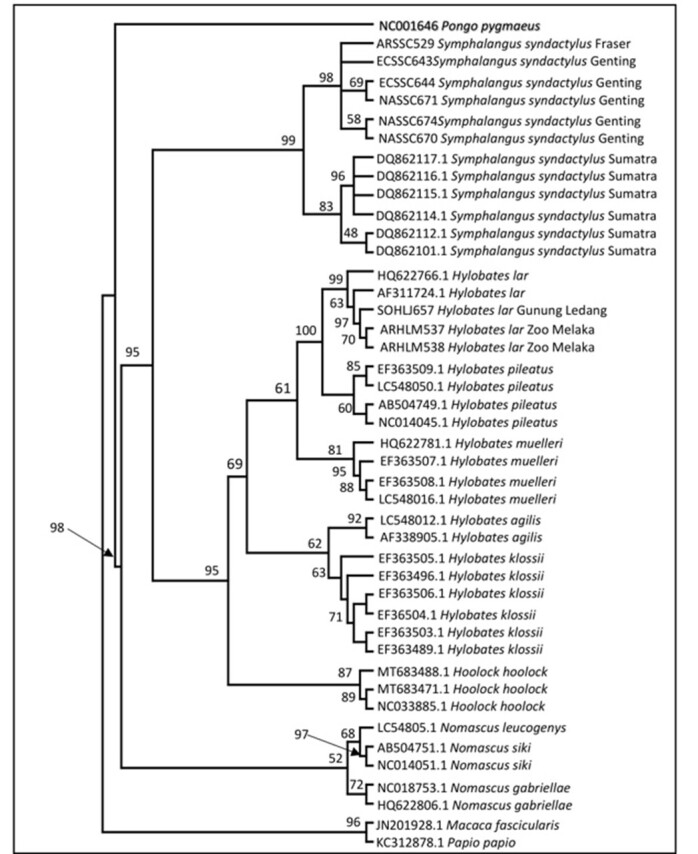
Maximum Likelihood (ML) phylogeny tree. Bootstrap values are indicated above the branch.

The ML tree (Fig. 4) supported the divergence between the two populations of *Symphalangus*. Within the *Symphalangus* major clade, two clades were formed, which were the Peninsular Malaysia Siamang clade (98% bootstrap value) and Sumatran Siamang clade (83% bootstrap value). The Sumatran clade had a typical branching pattern to the NJ and MP trees. However, in the Peninsular clade, four different branches were formed. The first branch, represented by NASSC670 and NASSC674, grouped together with a 58% bootstrap value. The second branch cladding of NASSC671 and ECSSC644 with a 69% bootstrap value, while ARSSC529 and ECSSC643 branched individually. The ML tree illustrated almost a typical branching pattern as NJ and MP analysis for hylobatids unless for the *Nomascus* position. A 98% bootstrap value supported the clustering of all Hylobatidae members. In contrast to NJ and MP, the *Nomascus* clade (52% bootstrap value) diverged from the *Symphalangus* + *Hoolock* + *Hylobates* clade (95% bootstrap value). Later, *Hylobates* and *Hoolock* were grouped in the monophyletic clade with a bootstrap value (95%) separated from the *Symphalangus* clade. This clade later diverged into two subclades represented by *Hylobates* and *Hoolock*, both of which were supported by 100% and 87% bootstrap values, respectively.

### Bayesian Inference tree

**Figure 5.**
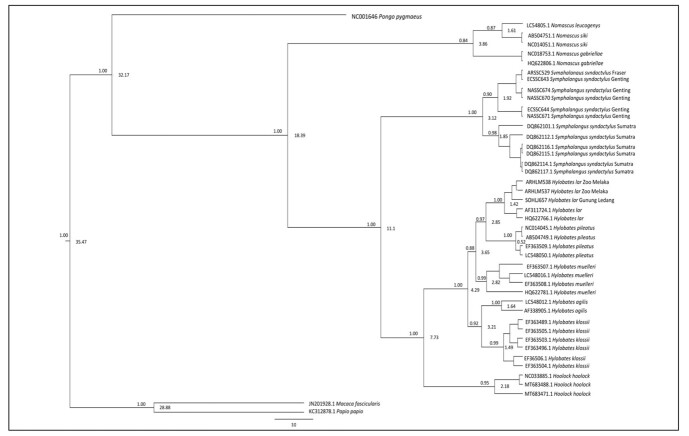
Summary of Bayesian inference and molecular clock phylogenetic tree. Values above the branch represent the posterior probability for BI. Values on the nodes represent the divergence times of the clade.

The BI tree (Fig. 5) agreed with the topological branch with ML analysis. A high posterior probability (PP) value (1.00) supported the grouping of all Hylobatidae genera in the same clade. However, in the Hylobatidae clade, *Nomascus* was recognised as a basal genus with 0.84 PP, which diverged the earliest and formed a sister clade to *Symphalangus*, *Hoolock* and *Hylobates*. Three remaining genera were grouped in the same clade with a 1.00 PP value. Then, it was followed by divergence of *Symphalangus* forming its own clade supported by 1.00 PP. *Hoolock* and *Hylobates* still branched together with 1.00 PP and later separated and formed a different clade with 0.95 and 1.00 PP values, respectively. Similar to other phylogenetic trees, BI showed absolute distinction of two clades of *Symphalangus* with high PP support. The first Siamang clade represented the Peninsular Malaysia Siamang population supported by 1.00 PP and the second clade comprised all Sumatran Siamangs with a 0.98 PP value. Within the Peninsular Malaysian clade, ECSSC644 and NASSC671 had the closest genetic relationships as a result of the NJ tree. For the Sumatran clade, the first individual to diverge was DQ862101.1, followed by DQ862112.1.

### Molecular clock estimation

The estimated evolutionary divergence dates were illustrated and supported by Bayesian analysis, as shown in Fig. 5. The analysis indicated that *S.syndactylus* diverged approximately 11.1 million years ago (MYA), splitting the species from the *Nomascus* clade. Later on, *S.syndactylus* was separated into two clades. The molecular clock separation between the Peninsular Malaysia and Sumatran populations of *S.syndactylus* occurred approximately 3.12 MYA. Splitting event occurred much earlier within the Peninsular Siamang population (1.92 MYA) than in the Sumatran Siamang population (1.85 MYA). The tree genus group of the family Hylobatidae (*Symphalangus*, *Hoolock*, *Hylobates* and *Nomascus*) diverged from *P.pygmaeus*, a split that was estimated to occur 32.17 MYA. *Nomascus* radiation occurred approximately 3.86 MYA, splitting from *Symphalangus, Hoolock* and *Hylobates* at 18.39 MYA. *Hoolock* and *Hylobates* diverged from *Symphalangus* 11.1 MYA. Then, this was followed by speciation of *Hylobates* in several events at 4.29 MYA. Amongst *Hylobates*, the clade formed by *H.lar*, *H.pileatus* and *H.muelleri* was the earliest group to branch out from the other *Hylobates* clades, which diverged approximately 3.65 MYA. Another lineage branched out and separated the clade (comprising two species, namely, *H.agilis* and *H.klossii*) approximately 3.21 MYA.

### Minimal spanning network

**Figure 6.**
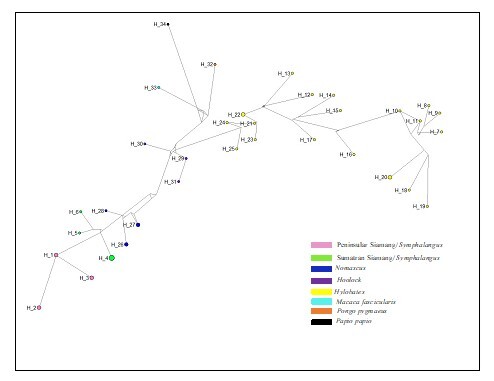
The minimum spanning network (MSN) illustrating the relationships between the *Symphalangus* population and other hylobatids. Each circle represents a haplotype.

MSN was used to visualise the associations between haplotypes, based on the haplotype analysis (Fig. 6). The network analysis revealed that the Peninsular Malaysia Siamang has a different haplotype from the Sumatran Siamang. *Symphalangus* contained six unique haplotypes: Hap_1 to Hap_3 characterised the haplotype of the Peninsular Siamang population and Hap_4 to Hap_6 represented the Sumatran Siamang population. The analysis depicted Hap_1 (Peninsular Malaysia Siamang) as the connecting point to Sumatran Siamangs with at least eight mutational steps as the fewest steps to indicate the relationship between these two populations. The MSN also revealed three haplotypes of *Nomascus* (Hap_26, Hap_27 and Hap_28), three haplotypes represented *Hoolock* (Hap_29, Hap_30 and Hap_31) and 18 haplotypes of *Hylobates* (Hap_12 to Hap_25) varied according to the species. The result depicted the fewest mutational steps (9) illustrated between *Symphalangus* and *Nomascus* haplotypes compared to other genera. Thus, we drew the closest relationship between *Symphalangus* and *Nomascus* amongst hylobatids. *Hoolock* and *Hylobates* haplotypes were adjacent to each other with at least 21 mutational steps.

## Discussion

### Phylogenetic relationship amongst hylobatids

Phylogenetic trees indicate the genetic relationships amongst the members of Hylobatidae. Two different branch topologies of the hylobatid clade were obtained to explain the genetic relationships amongst these genera. In the NJ and MP trees, *Nomascus* and *Symphalangus* branched together, forming a sister clade to the *Hylobates*–*Hoolock* clade. For ML and BI trees, alternatively exclude *Nomascus* from being in a clade together with *Symphalangus*, which formed a monophyletic clade with *Hoolock* and *Hylobates*. Moderate support in bootstrap value was recorded for the topology of the *Nomascus*–*Symphalangus* clade, but there was a high support for the *Hylobates*–*Hoolock* clade formation. However, the PP value showed positive support for each topography clade formed amongst the hylobatids. The *Symphalangus* position is prone to form a clade together with *Hylobates* + *Hoolock* in bootstrap analysis, specifically NJ and MP trees. This can be explained by the fact that the earliest primatologists classified *Symphalangus* as a subgenus of *Hylobates* (Kloss 1929). Regardless, Kloss (1929) discovered that the dwarf gibbon is the absolute intermediate species between ordinary gibbons and siamangs, excluding *Symphalangus* as an out-group and grouping all gibbons into one genus. This is parallel to this study as *Symphalangus* later diverged from the Hylobates clade. In addition, the results showed high support of bootstrap and PP values for the branching of *Hylobates* and *Hoolock* in the monophyletic clade (without *Symphalangus* and *Nomascus*).

Hoolock gibbons were previously classified as members of the genus *Bunopithecus* (Prouty et al. 1983) and *Hylobates* and *Hoolock* once formed a monophyletic clade, as proposed by Groves (2001) and Brandon-Jones et al. (2004). Mootnick and Groves (2005) reclarified the Hoolock gibbons as having different karyotypes from *Hylobates*, renaming the genus as *Hoolock*. Thus, this study supports the distinction of *Hoolock* from *Hylobates* as mentioned by a previous researcher. Groves (2001) acknowledged *Nomascus* as a subgenus of *Hylobates*, which contradicted our study, which suggested *Nomascus* as the basal genus of hylobatids. However, because of contrasting support between bootstrap and PP values in this study, the relationship of these four genera to each other remains obscure as no previous research revealed typical branching patterns (Carbone et al. 2014). The relationships amongst the four genera were previously unresolved, despite the efforts of experts, such as Chan et al. (2010) and Matsudaira and Ishida (2010), who successfully constructed the evolutionary tree that supported *Nomascus* as the pioneer genus (without adding *Hoolock*), but the structure collapsed when all genera were analysed (Roos 2016).

However, the current classification of *Nomascus* as a basal taxon of Hylobatidae has been accepted, as illustrated in the Maximum Likelihood tree by Israfil et al. (2011), which is consistent with our study with stronger support as more samples of hylobatids were included. The groupings of different *Hylobates* species (*H.lar*, *H.muelleri*, *H.agilis*, *H.pileatus* and *H.klossii*) are congruous for all four phylogenetic trees. According to Roos (2016), the branching pattern amongst *Hylobates* species may be controversial, but there is a common agreement on *H.pileatus* forming the basal lineage of this group. However, our study indicates contradictory results, where *H.klossii* and *H.agilis* (in a monoclade) diverged the earliest, followed by the divergence of *H.muelleri*, *H.lar* and *H.pileatus*. The relationships between the species comprising clades *H.klossii–H.agilis* and *H.lar–H.muelleri* remain unresolved as both bootstrap and PP values indicated low support, which agrees with the results published by Carbone et al. (2014). This ambiguity is common in situations where sympatric species may migrate, leading to hybridisation (Roos 2016).

### Radiationship of the Siamang population

In terms of divergence times, the Hylobatidae mitochondrial *D*-loop sequences estimate divergences in this study is slightly near to those proposed by Israfil et al. (2011) and these occurred approximately 19.7–24.1 MYA. However, the divergence of *Syndactylus* in this study did not match the divergence date of *Hylobatessyndactylus* from *Nomascus*, estimated to be approximately 5.6–7.2 MYA as proposed by Israfil et al. (2011). This mismatch in divergence age might be due to the high number of samples and the type of locus used. This study only covered a part of the mitochondrial region, which is the D-loop, compared with Israfil et al. (2011), who used the whole mitochondrial and sex-linked gene. However, the D-loop region is still considered more relevant to the phylogenetic study of rapidly changing gene that radiate over a short period of time (Abdul-Latiff et al. 2019). In addition, different topological branches elucidate the different time ranges, as Israfil et al. (2011) proposed *Hoolock* as the basal genus instead of *Nomascus*.

The results connotated Siamangs from Peninsular Malaysia as the basal taxon of radiation because the population divergence date was much earlier than that of Sumatran Siamangs. This is comparable to studies on *Trachypithecuscristatus* (Roos et al. 2008) and *Hylobates* (Whittaker et al. 2007, Israfil et al. 2011) that the mainland population is considered as ancestral stock, while the southern population is derived species. Thus, it can be presumed that the speciation of Siamangs occurred first in Peninsular Malaysia, after which Siamangs migrated and diversified in Sumatra. As argued by Chatterjee (2006) and Chatterjee et al. (2009), *Hylobates* radiated from mainland Southeast Asia southwards and eastwards (via Peninsular Malaysia and Sumatra) ~ 8.3 MYA. During the Pleistocene, the early diversification of hylobatids was followed by a period of rapid speciation, notably within the concolour and lar gibbon genera (Carbone et al. 2014). *Symphalangus* was claimed to have diverged from the hylobatid lineage through southward expansion (Chatterjee et al. 2009) as it can be traced in the west of the Mekong River (Thinh et al. 2010).

Past scenarios might have caused the small population of different species to thrive in small refugees during the Pliocene (~ 3.17–0.31 MYA), which consequently drove the species apart and formed different populations (Wang 1994). At this age, *Presbytis* and *Macaca* had colonised Sumatra from the Malay Peninsula throughout the Late Pliocene and Early Pleistocene, where cold climate occurred at approximately 1.8 Ma (Harrison et al. 2006). Later, sea level falls formed a route that connected the Javan and Peninsular Malaysia mainland first, without connecting to Sumatra and Borneo during the Pleistocene (~ 1.25 MYA) (Hall et al. 1998). This was followed by drastic falling of sea levels (Gorog et al. 2004) causing the mainland to expand and being connected to Sumatra (Roos et al. 2008). The connection between these two regions via a land corridor has allowed the radiation of *Hylobates* (Chatterjee 2006) and *T.cristatus* (Roos et al. 2008) from the mainland to the southward region. Thus, we deduced the baseline of the same land bridge that caused the migration of Siamangs from Peninsular Malaysia to Sumatra.

Compared to this study, splitting of Siamangs occurred 3.12 MYA, the age of which is in line with the late Pliocene era when Siamang from Peninsular might move along with *Presbytis* and *Macaca* migrating from Malay Peninsular to Sumatra where Peninsular Malaysia and Sumatra were already connected to each other. It is possible Siamang that colonised Peninsular Malaysia migrate after a vicariance event such as the rise and fall of sea level (Abdul-Latiff et al. 2014) resulting in the existence of Siamang in Sumatra. Thus, we proposed the Peninsular Malaysia Siamang (*S.s.continentis*) positioned in the mainland as the ancestor and the Sumatran Siamang (*S.s.syndactylus*) that diverged later as derived species.

### Taxonomic position of the Symphalangus subspecies

On the basis of the previous result, the taxonomic position of the two proposed subspecies is accepted because the *D*-loop analysis illustrated significant subspecies distinction. Raffles (1821) was the first to describe *Symphalangus*, based on the sample found in Sumatra. At that point, he concluded that the Siamang was a single species and no further classification was made. None of the fossil records was reviewed to clarify the ages of the Siamang population in Peninsular Malaysia and Sumatra to confirm which population existed the earliest. However, Thomas (1908) compared Sumatran skulls with British Museum skull specimens to explore the proclivity for minor variations in Peninsula specimens. This result indicates that the skull size of the Peninsula Siamang is smaller than that of the Sumatran Siamang. Thomas (1908) referred to Siamangs from Peninsular Malaysia as *S.s.continentis* and Sumatran Siamangs as *S.s.syndactylus.*

The subspecies distinction was embraced by another morphological description, recorded by Kloss (1929). In terms of colouration, the face of *S.s.syndactylus* is loosely covered with white fur and faintly tinted with brown, whereas *S.s.continentis* has less pale hair on the face and fur that is frequently less thick than that of Sumatran Siamangs, which invade colder climates (Kloss 1929). Both populations are physically comparable in terms of size, although *S.s.continentis* seems larger than *S.s.syndactylus* (*Kloss 1929*). The morphological characteristics in terms of skull size (Thomas 1908) and body size (Kloss 1929) for each subspecies seem to be contradicting. It cannot be confirmed that the skull size of *S.s.syndactylus* is larger than that of *S.s.continentis* as described by Thomas (1908) because no evidence has been provided on which sex of Siamangs for each subspecies has been used for comparison. This is because different skull sizes have been documented for different sexes as indicated by island rule (Welch 2009). Hooijer (1960) argued that the size and proportions of the skulls of *S.s.syndactylus* are remarkably varied in males and females. The average male skull is larger than the female skull with a thinner brain case, more projected orbiting and deeper postorbital constriction. *Symphalanguss.continentis* also has sexual variations of the skull that are comparable to those of *S.s.syndactylus* (Hooijer 1960).

**Figure 7.**
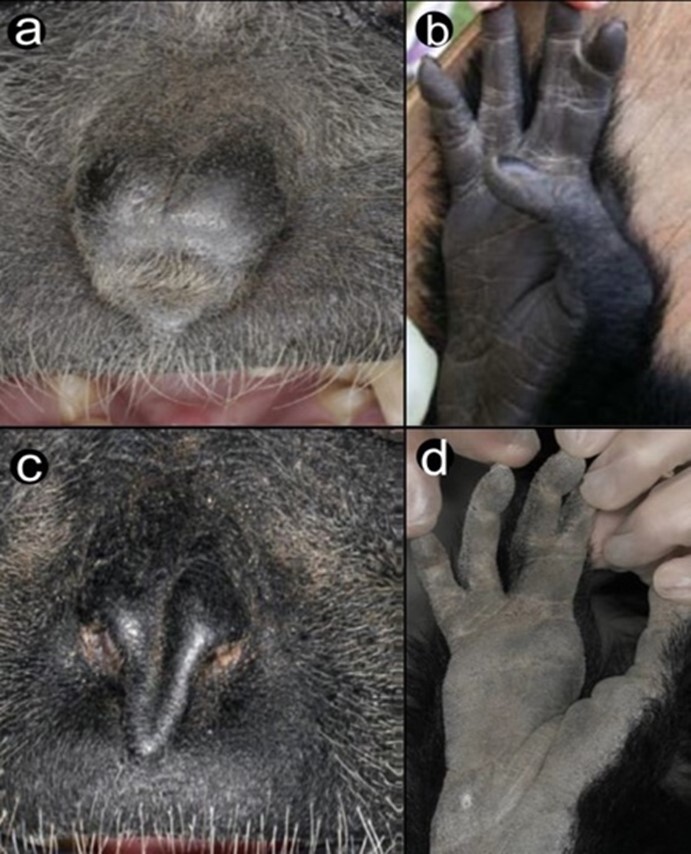
*Symphalanguss.continentis* (a and b) and *S.s.syndactylus* (c and d) nose structure and toe structure (Mootnick 2006).

Hylobatids have been postulated to be a mechanism for decrease in size or dwarfing (Pilbeam 1996). Body size changes in response to a colonising continental species may be adaptable to an island depending on the island mass and its existing animals and vegetation (Welch 2009, Burness et al. 2001). *Hylobates* have evolved with specific features, such as a reduced body size and well-developed fore-limb suspensory locomotion (Reichard et al. 2016). However, Siamangs are enigmatic as their size is one-half to two times larger than that of other gibbons. More recently, other morphological features, such as nose and toe structures (Fig. 7), were reported by Mootnick (2006). *Symphalangus* *s.syndactylus* has a shallow and long tapering point pattern over its nostrils and webbing between the fourth and fifth toes, whereas *S.s.continentis* has a wider nose and second and third toes extending up to the distal interphalangeal joint (Mootnick 2006).

These features add evidence for classifying the Peninsular Malaysian Siamangs as *S.s.continentis* and Sumatran Siamangs as *S.s.syndactylus.* Other researchers have continued to classify Siamangs according to morphological appearance as the molecular approach requires genetic data that were previously unavailable, thus hindering molecular studies at the species level (Roos 2016). However, this study successfully reclarified the existence of two subspecies of *Symphalangus*.

### Siamang conservation

The primary threat to Siamangs is forest fragmentation, which limits their ranging area and the availability of food sources (Marshall 2009). As with other primates, the Siamang habitats in Malaysia are being disrupted either directly or indirectly by human activities. For instance, in a survey of 14 forest fragments that include the Virgin Jungle Reserves and adjacent disturbed forests in Peninsular Malaysia, *S.syndactylus* was present in two out of six large fragments and absent from all small- and medium-sized forest fragments (Laidlaw 2000). Habitat fragmentation leads to an increased risk of predation and hunting of this species, resulting in an increased probability of local extinction. To protect this species, both *in situ* and *ex situ* conservation are needed. Molecular studies on Siamangs to reveal taxonomical classification have shown that Peninsular and Sumatran Siamangs have different gene pools. It is important to acknowledge the genetic relationship up to the subspecies level as this may affect the decision for translocation as similar gene pools should be grouped together to encourage survival. Our results confirmed subspecies classifications within *S.syndactylus* and this information will be useful in identifying the most suitable region for Siamang translocation for the purpose of conservation.

## Conclusions

Mitochondrial *D*-loop region analysis confirmed the subspecies within *S.syndactylus*, *S.s.continentis* is found in Peninsular Malaysia, whereas *Symphalangus* *s.syndactylus* inhabits Sumatra, Indonesia. Sequence analysis also confirmed the phylogenetic relationships proposed in previous studies regarding *Hylobates*. Our study updated the taxonomic classification of Siamangs and the members of Hylobatidae. Such genetic identification is crucial to Siamang conservation because it enables the allocation of each individual to the most suitable population and habitat. Primates that are introduced to the right population have a higher chance of survival.

## Figures and Tables

**Table 1.** List of faecal genetic samples and DNA sequences from GenBank.

**No**	**Sample**	**Species**	**Origin**
1	ECSSC643	*S.s.continentis*	Genting Highlands, Pahang
2	ECSSC644	*S.s.continentis*	Genting Highlands, Pahang
3	NASSC670	*S.s.continentis*	Genting Highlands, Pahang
4	NASSC671	*S.s.continentis*	Genting Highlands, Pahang
5	NASSC674	*S.s.continentis*	Genting Highlands, Pahang
6	ARSSC529	*S.s.continentis*	Fraser’s Hill, Pahang
7	DQ862101.1	*S.s.syndactylus*	Sumatra Lappan (2007)
8	DQ862112.1	*S.s.syndactylus*	Sumatra Lappan (2007)
9	DQ862114.1	*S.s.syndactylus*	Sumatra Lappan (2007)
10	DQ862115.1	*S.s.syndactylus*	Sumatra Lappan (2007)
11	DQ862116.1	*S.s.syndactylus*	Sumatra Lappan 2007
12	DQ862117.1	*S.s.syndactylus*	Sumatra Lappan (2007)
13	ARHLM537	*Hylobateslar*	Zoo Melaka
14	ARHLM538	*H.lar*	Zoo Melaka
15	SOHLJ657	*H.lar*	Gunung Ledang, Johor
16	AF311724.1	*H.lar*	Roos and Geissmann (2001)
17	HQ622766.1	*H.lar*	Chan et al. (2010)
18	HQ622781.1	*H.muelleri*	Chan et al. (2010)
19	EF363508.1	*H.muelleri*	Whittaker et al. 2007
20	EF363507.1	*H.muelleri*	Whittaker et al. (2007)
21	LC548016.1	*H.muelleri*	Matsudaira and Ishida (2010)
22	LC548012.1	*H.agilis*	Matsudaira and Ishida (2010)
23	AF338905.1	*H.agilis*	Andayani et al. (2001)
24	EF363506.1	*H.klossii*	Whittaker et al. (2007)
25	EF363504.1	*H.klossii*	Whittaker et al. (2007)
26	EF363505.1	*H.klossii*	Whittaker et al. (2007)
27	EF363503.1	*H.klossii*	Whittaker et al. (2007)
28	EF363489.1	*H.klossii*	Whittaker et al. (2007)
29	EF363509.1	*H.pileatus*	Whittaker et al. (2007)
30	EF363496.1	*H.pileatus*	Whittaker et al. (2007)
31	LC548050.1	*H.pileatus*	Matsudaira and Ishida (2010)
32	AB504749.1	*H.pileatus*	Matsudaira and Ishida (2010)
33	NC014045.1	*H.pileatus*	Unpublished
34	MT683471.1	*Hoolockhoolock*	Trivedi et al. (2021)
35	MT683488.1	*H.hoolock*	Trivedi et al. (2021)
36	NC033885.1	*H.hoolock*	Unpublished
37	AB504751.1	*Nomascussiki*	Matsudaira and Ishida (2010)
38	NC01451.1	*N.siki*	Unpublished
39	LC548051.1	*N.leucogenys*	Matsudaira and Ishida (2010)
40	NC018753.1	*N.gabriellae*	Chan et al. (2010)
41	HQ622806.1	*N.gabriellae*	Chan et al. (2010)
42	NC001646	*Pongopygmaeus*	Horai et al. (1992)
43	JN201928.1	*Macacafascicularis*	Unpublished
44	KC312878.1	*Papiopapio*	Ferreira da Silva et al. (2014)
